# Irradiated riboflavin over nonradiated one: Potent antimigratory, antiproliferative and cytotoxic effects on glioblastoma cells

**DOI:** 10.1111/jcmm.18288

**Published:** 2024-04-10

**Authors:** Sedat Kacar, Ceyhan Hacioglu, Fatih Kar

**Affiliations:** ^1^ Department of Histology and Embryology, Faculty of Medicine Eskisehir Osmangazi University Eskisehir Turkey; ^2^ Department of Surgery, Division of Oncologic Surgery Indiana University School of Medicine Indianapolis Indiana USA; ^3^ Department of Medical Biochemistry, Faculty of Medicine Duzce University Duzce Turkey; ^4^ Department of Biochemistry, Faculty of Medicine Kutahya Health Sciences University Kutahya Turkey

**Keywords:** apoptosis, caspase 3, 7 and 9, glioblastoma cells, inflammation, metalloproteinases, oxidative stress, riboflavin, SIRT1

## Abstract

Riboflavin is a water‐soluble yellowish vitamin and is controversial regarding its effect on tumour cells. Riboflavin is a powerful photosensitizer that upon exposure to radiation, undergoes an intersystem conversion with molecular oxygen, leading to the production of ROS. In the current study, we sought to ascertain the impact of irradiated riboflavin on C6 glioblastoma cells regarding proliferation, cell death, oxidative stress and migration. First, we compared the proliferative behaviour of cells following nonradiated and radiated riboflavin. Next, we performed apoptotic assays including Annexin V and caspase 3, 7 and 9 assays. Then we checked on oxidative stress and status by flow cytometry and ELISA kits. Finally, we examined inflammatory change and levels of MMP2 and SIRT1 proteins. We caught a clear antiproliferative and cytotoxic effect of irradiated riboflavin compared to nonradiated one. Therefore, we proceeded with our experiments using radiated riboflavin. In all apoptotic assays, we observed a dose‐dependent increase. Additionally, the levels of oxidants were found to increase, while antioxidant levels decreased following riboflavin treatment. In the inflammation analysis, we observed elevated levels of both pro‐inflammatory and anti‐inflammatory cytokines. Additionally, after treatment, we observed reduced levels of MMP2 and SIRT. In conclusion, radiated riboflavin clearly demonstrates superior antiproliferative and apoptotic effects on C6 cells at lower doses compared to nonradiated riboflavin.

## 1 INTRODUCTION

Based on GLOBOCAN database, cerebral and nervous system malignancies ranked 12th in terms of mortality among 33 different cancer categories in 2020, with 251,329 reported deaths and a reported incidence of 308.102 new cases. Notably, the Asian region accounts for around half of these death and incidence rates, with Europe coming in second.^1^ Gliomas are the most common tumours in the central nervous system among cancers of the brain and nervous system. The glioblastoma, also known as glioblastoma multiforme, is the most aggressive type of glioma that arises from astrocytes. Multiple cellular models are available to comprehend the biology of glioma cells. One of the most common ones is C6 glioblastoma cells. C6 glioblastoma cells were first obtained by Benda et al. (1968) from the brain tissues of Wistar Furth strain rat with N‐nitroso methyl urea‐induced glioblastoma.^2^

Riboflavin is a water‐soluble yellowish vitamin. It exists in the two coenzymatic forms—flavinadenine dinucleotide and flavinadenine mononucleotide. In literature, contradictory data regarding riboflavin's effect on cancer exist. Riboflavin (Vitamin B2) was discovered to exhibit tumour‐inhibiting properties on pro‐monocytic lymphoma cells with the decreased migration in cells.^3^ However, in the same article, apoptosis or cell death by necrosis were not detected after riboflavin treatment (250 μg/mL ~ 665 μM) and the distribution of live and dead cells was similar to that of untreated cells. On the contrary, riboflavin is also reported to boost cancer growth, invasiveness and tumour survival. In addition, the suppression of flavin containing enzymes is documented to arrest cancer development.^4^, ^5^ Therefore, exploring riboflavin's possible uses and consequences in cancer‐related research and therapies requires a cautious and nuanced approach due to the complicated and conflicting functions seen in the interplay between riboflavin and cancer. Moreover, riboflavin is recognized as a potent photosensitizer that generates reactive oxygen species (ROS) under photoexcitation. Its efficacy as a drug in photodynamic treatment for malignancy removal stems from these photosensitizing properties. Upon exposure to radiation, riboflavin undergoes intersystem conversion with molecular oxygen, leading to the production of ROS.^6^ This characteristic enhances its potential to exert antiproliferative and apoptotic effects on cancer cells. Previous investigations have documented that irradiated riboflavin exhibits a robust antitumour impact on HL60 leukaemia cells, along with antiproliferative and antimetastatic effects on solid tumours.^7^ Furthermore, there is evidence suggesting that the molecular mechanism underlying the antitumour properties of irradiated riboflavin is grounded in the modulation of various proteins, leading to the induction of apoptosis and increased aggressiveness in cancer cells.^8^

In this study, we aim to investigate the impact of both irradiated and nonirradiated riboflavin. Subsequently, our analysis will focus specifically on irradiated riboflavin, exploring aspects such as the mode of cell death, inflammation, metalloproteinase activity, as well as specific activation and oxidative stress responses.

## 2 MATERIALS AND METHODS

### 2.1 Cell cultivation

C6 cells are gained from the ATCC—American Type Culture Collection—(RKV, Maryland, US), and grown in DMEM, including 10% (v/v) of FBS and 1% (v/v) of antibiotics (penicillin–streptomycin) in 5% CO_2_ incubator fixed at 37°C. During experiment, the cells were grown in 25 cm^2^ flasks, allowed to attach and have connections with each other to grow and stabilize faster. Then, they are transferred to corresponding plates for subsequent experiments such as 75 cm^2^ cell culture flasks or 24‐well plates etc. Every 3 days cell medium is replaced with fresh one and before 80% confluence, cells are passaged and reseeded to new flasks.

### 2.2 Riboflavin irradiation

Irradiation of riboflavin is carried out according to previous methods.^9^ Briefly, riboflavin (4000 μM) is dissolved in DMEM and the solution is maintained in a Petri dish and exposed to UV light (254 nm, 40 W/cm^2^) for 30 min with UV lamp placed 40 cm close. Riboflavin solution not exposed to UV is used as nonradiated riboflavin.

### 2.3 MTT cytotoxicity assay

MTT or 3‐(4,5‐dimethylthiazol‐2‐yl)‐2,5‐diphenyltetrazolium bromide is a commonly used cytotoxicity test that relies on the MTT reduction of live cells, which is performed by the mitochondrial enzymes, leading to the formation of formazan salts.^10^ Afterward, the formazan salts formed were dissolved using suitable solvents. The C6 cell line was introduced into 96‐well plates for the study, with a population of 5000 cells per well. Either nonradiated and irradiated riboflavin was given to the cells in a serially diluted manner, spanning a concentration range from 0 to 1000 μM, after allowing the cells to adhere to the plate's bottom. Initial treatments helped define the riboflavin concentration range. The matching treatment period (24, 48 or 72 h) was followed by the removal of the cell media from the wells. Then, the wells were filled with 0.5 mg/mL MTT, and the cells were placed in an incubator for a duration of 4 h at 37°C under a 5% CO_2_ atmosphere. After the incubation, 100 μL of dimethylsulfoxide was added to the wells to solubilize the formazan salts formed. Lastly, the optical densities were gauged at the wavelength of 570 nm using BioTek 800 TS Absorbance Reader (Winooski, VT, US). The relative viability percentages of cells administered with riboflavin were computed using the viability of control‐not treated‐cells, which was assumed to be 100% viable. The following formula was employed to determine the percentages of cell viability^11^, ^12^:

$\begin{array}{c} \left[\left(\text{riboflavin}-\text{treated cells’ absorbance}-\text{blank’s absorbance}\right)\right./ \end{array}$ $\begin{array}{c} \left.\left(\text{control’s average absorbance}-\text{blank’s absorbance}\right)\right]\times 100 \end{array}$

We continued the upcoming experiments by selecting three concentrations of radiated riboflavin: (1) 31.25 μM: the concentration of irradiated riboflavin where a first statistically significant decrease in cellular viability was spotted, (2) 62.5 μM: the concentration of irradiated riboflavin dose that is around IC50 and (3) 93.75 μM: the sum of first and second doses mentioned above. We opted not to proceed with the dose of 125 μM due to its high toxicity to the cells. Using this concentration would not allow for a meaningful evaluation of the effects of substance as it led to cell death.

### 2.4 Cell protein extraction

Protein isolates were utilized for conducting various assays, including sirtuin 1 (SIRT1), matrix metalloproteinase 2 (MMP2), tumour necrosis factor alpha (TNF‐α), interleukin 10 (IL‐10), interleukin 1β (IL‐1β), total oxidant status (TOS), total antioxodant capacity (TAC), caspase 3 and 9 assays. Three different doses of radiated riboflavin (31.25, 62.5 and 93.75 μM) were exploited in upcoming tests. C6 cell line, cultivated in the flasks of 75 cm^2^, was allotted into four groups: untreated or treated with 31.25, 62.5 and 93.75 μM concentrations of riboflavin for 24 h. Once treatment is over, the cells were gently rinsed with ice‐cold PBS (pH = 7) and detached using trypsin, centrifuged at 300 *g* for 15 min at 4°C. The sample's liquid portion was discarded, and 500 mL of cold RIPA lysis solution containing a protein inhibitor cocktail was used to lyse the cell pellets. To eliminate cell debris, the lysates were centrifuged at 16000 *g* for 20 min at 4°C after being intermittently vortexed on ice for 30 min. The biuret test was utilized for protein quantification.^13^ The poised protein isolates were immediately exploited in the subsequent tests.

### 2.5 Sirtuin 1 assay

Sirtuin 1 amounts were gauged exploiting a comercially available ELISA set (Cloud‐Clone Corp., Texas, USA, SEE912Ra) using a plate reader. The quantification of SIRT1 from protein isolates was done by referencing the plot prepared with known optical densities. The outcomes were expressed as ng/mL.

### 2.6 MMP2 measurement

Matrix metalloproteinase 2 (MMP2) amounts were gauged exploiting a commercially available ELISA set (Cloud‐Clone Corp., Texas, USA, SEA100Ra) using a plate reader. The quantification of MMP2 from protein isolates was done by referencing the plot prepared with known optical densities. The outcomes were expressed as ng/mL.

### 2.7 The measurements of inflammation‐associated cytokine levels

The amounts of IL‐10 and TNF‐α were gauged exploiting commercial kits (Sigma Aldreich Corp, MO, USA, Cat no: RAB0480‐1KT and RAB0247‐1KT) from cell homogenenate samples in a plate reader. The levels of IL‐1β are were gauged exploiting a commercially available kit (Thermo Fischer Scientific, MA, USA, Cat no: ERIL1B) in A plate reader. The quantification of IL‐10, TNF‐α and IL‐1β from protein isolates was done by referencing the plot prepared with known optical densities. The outcomes were expressed as ng/mL.

### 2.8 Caspase 3/9 activities

The levels of caspase 3 were gauged exploiting commercially available kits (Cloud‐Clone Crorp, MO, USA, SEA626Ra) in a plate reader. The caspase 9 amountswer e gauged exploiting a comercially available kit (MyBioSources Biotech. Com., CA, USA, MBS1602954) in a plate reader. The caspase 3 and caspase 9 amounts in protein isolates were quantified by referencing the plot prepared with known optical densities. The outcomes were expressed as ng/mL.

### 2.9 TAC and TOS levels

The levels of serum total antioxidant capacity (TAC, Cat. No: RL0024) and total oxidant status (TOS, Cat. No: RL0017) were assessed using comercially available kits (Rel‐Assay Diagnostics, Sehitkamil, Gaziantep, Turkey) from cell homogenates.

### 2.10 Annexin V Assay by flow cytometry

The apoptotic impact of the radiated riboflavin on C6 cells was assessed using the Muse Annexin V kit of Merck Millipore. The C6 cell line was grown in 24 wells including plates; split into four groups: either not treated or treated with 31.25, 62.5 and 93.75 μM of radiated riboflavin. Following a 24‐h treatment period, the cell culture media was disposed, and the cell line was rinsed using PBS. Thereafter, the cell line was washed with EDTA‐containing PBS and maintained in trypsin–EDTA for 3–5 min. The detached cells were exposed to 10 min‐centrifugation at 150 *g*, and the pellet was then reconstituted with new medium. In the meantime, 100 μL of solution of annexin V plus propidium iodide (provided with the kit) were combined in an Eppendorf tube. The produced cell suspension was then introduced at a volume of 100 L into this mixture. These two solutions were incubated at room temperature for 20 min in the dark. Finally, the cells were analysed exploiting the Muse TM Cell Analyser (Merck Millipore, Hayward, CA, USA).

### 2.11 Caspase 3 and 7 assay by flow cytometry

The activities of caspase 3 and 7 enzymes of the radiated riboflavin on C6 cells were assessed using the Muse™ Caspase‐3/7 kit of Merck Millipore. The cell line was grown in 24 well containing plates and split into four groups: either not treated or treated with irradiated riboflavin at 31.25 μM, 62.5 μM and 93.75 μM of radiated riboflavin. Upon the treatment period is over and the cells are dislodged off the flasks, the caspase 3/7 reagent provided in the kit was diluted with phosphate‐buffered saline (PBS) at a ratio of 1:8, resulting in the caspase working solution. Concurrently, 148 μL of buffer solution was combined with 2 μL of the cell death marker 7‐AAD to form the working solution. Following this, the suspended cells were mixed with working solution with 10:1 ratio and left to 30 min‐incubation at 37°C. Finally, the cells were analysed exploiting the Muse TM Cell Analyser (Merck Millipore, Hayward, CA, USA).

### 2.12 Oxidative stress measurement by flow cytometry

The flow cytometric oxidative stress evaluation of the radiated riboflavin on C6 cells was assessed using the Muse Oxidative Stress Kit of Merck Millipore. The cell line was grown in 24‐well containing plates and split into four groups: either untreated or treated with irradiated riboflavin at 31.25 μM, 62.5 μM and 93.75 μM of radiated riboflavin. Once cells were treated and detached, the resultant pellet was then reconstituted in the supplemented buffer in the kit. Then the buffer solution was mix with the oxidative stress reagent with the ratio of 1:100. This mixture was further diluted with the buffer solution with the ratio of 1:80. To create the oxidative stress working solution, then, a mixture of 10 L of cells and 190 L of the prepared working solution was left to a 30‐min incubation at 37°C. Finally, the cells were analysed exploiting the Muse TM Cell Analyser (Merck Millipore, Hayward, CA, USA).

### 2.13 Statistical analysis

The statistical analysis of the data for caspase 3, caspase 9, sirtuin 1, cytokine measurements and inflammation‐related markers was conducted using the pre‐established statistical package of GraphPad and utilizing IBM SPSS Statistics, Version 29.0.1.0. Initially, the experimental data were assessed for normal distribution using the Shapiro–Wilk normality test. For normally distributed data, one‐way ANOVA was employed, and post hoc analysis was performed using Tukey's test. For the data of cell viability to figure out deviation between nonradiated and radiated riboflavin groups, a 2‐way ANOVA test is applied. The data were deemed as the mean ± standard deviation (SD). A *p*‐value less than 0.05 was accepted as statistically significant.

## 3 RESULTS

### 3.1 Cell viability assay results

**FIGURE 1:**
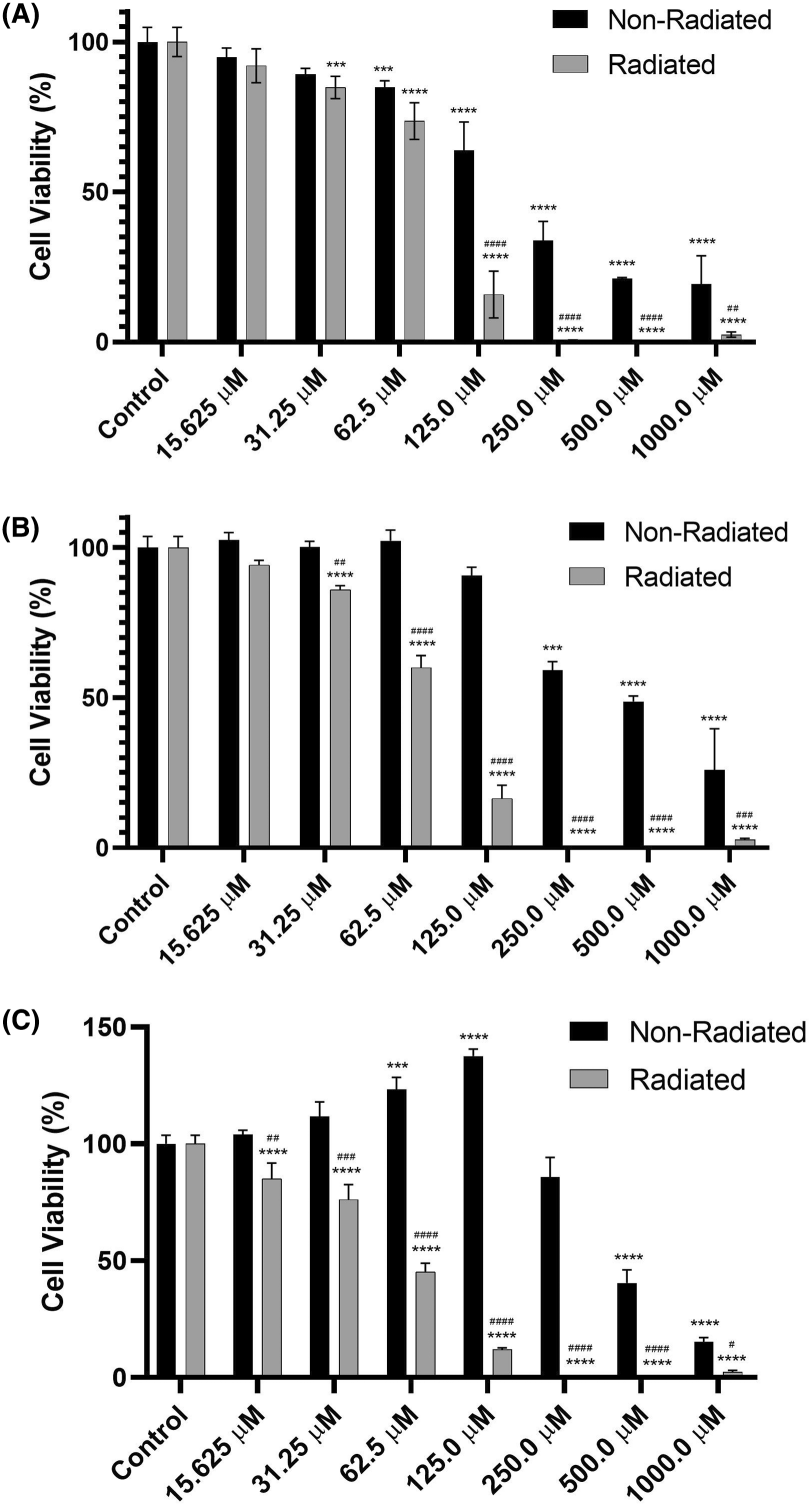
Cell viability graphs of nonradiated and radiated riboflavin‐administered C6 cell line for 24 (A), 48 (B) and 72 h (C). ***p < 0.001 and ****p < 0.0001 when compared to the respective untreated group. ###*p* < 0.001 and ^####^ *p* < 0.0001 when compared to same dose of nonradiated group. The outcomes are deemed as the average ± SD.

Three‐time courses—24, 48 and 72 h—were used in the MTT assay. In each time course, we employed seven different either nonradiated or radiated riboflavin concentrations including 7.8, 15.6, 31.3, 62.5, 125, 250 and 500 μM as well as untreated control. According to MTT results of 24 h nonradiated riboflavin treatment, no significant deviation was observed at the doses of 15.625 and 31.25 μM in comparison to the control levels (all *p* > 0.05). However, a significance deviation was detected at 62.5 μM, in which the viability was approximately 85%, (*p* < 0.001). The viability further diminished to 63.9% (*p* < 0.0001) at 125 μM, Finally, the viability was maintained between 33% and 19% (*p* < 0.0001) even at the concentrations greater than 125 μM (250, 500 and 1000 μM). Still viable cells were visible. On the contrary, according to MTT results of 24 h radiated riboflavin treatment, first dose where a significant change was detected was 31.25 μM (*p* < 0.0001), where the cell viability was 84.8. The cell viability reduced to 73.6% at 62.5 μM and sharply dropped to 15.8% at 125 μM. Discernibly, viability was hardly detectable at the concentrations greater than 125 μM (250, 500 and 1000 μM). As for comparison between nonradiated versus radiated riboflavin groups at the same dose group, a significant deviation was observed at 125 μM and above doses (Figure 1A).

The MTT results after 48 h of riboflavin treatment without radiation exposure do not indicate a significant result between control and treated cells up to 250 μM (*p* < 0.001), where cell viability was 59.2%. At the higher doses of 500 and 1000 μM, the viability further decreased to 48.7% and 25.8%, respectively. Even the cell viability was higher when compared to 24 h treatment. On the contrary, based on the MTT results from a 48 h of radiated riboflavin treatment, first dose where a significant change was detected was again 31.25 μM (*p* < 0.0001) like in 24 h treatment, where the cell viability was 86.0%. The cell viability reduced to 60.0% at 62.5 μM and sharply dropped to 16.4% at 125 μM. Similar to 24 h treatment, viability was hardly detectable at the concentrations greater than 125 μM (250, 500 and 1000 μM). The significant difference was seen at the dose as low as 31.25 μM and above between nonradiated and radiated riboflavin treated cells (Figure 1B).

According to the MTT results after 72 h of riboflavin treatment without radiation exposure, there was no significant difference between control and treated cells at the concetrations of 15.625 and 31.25 μM. Intriguingly, a significant increase (a possible proliferation) was detected at the doses of 62.5 μM (*p* < 0.001) and 125 μM (*p* < 0.0001). This increasing trend ceased at 250 μM, which showed no significant decrease from control. Then, the viability decreased to 40.5% and 15.3% at the doses 500 and 1000 μM, respectively. On the contrary, based on the MTT results from a 72 h of radiated riboflavin treatment, first dose where a significant change was as low as 15.625 μM—lowest dose used (*p* < 0.0001), where the cell viability was 85.0%. The cell viability reduced to 76.1% at 31.25 μM, 45.1 μM at 62.5 μM and sharply dropped to 12.1% at 125 μM. Similar to 24 and 48 h treatments, cell viability was hardly detectable at the concentrations greater than 125 μM (250, 500 and 1000 μM). The significant difference was seen at the dose as low as 15.625 μM and above between nonradiated and radiated riboflavin treated cells (Figure 1C).

### 3.2 Annexin V test

**FIGURE 2:**
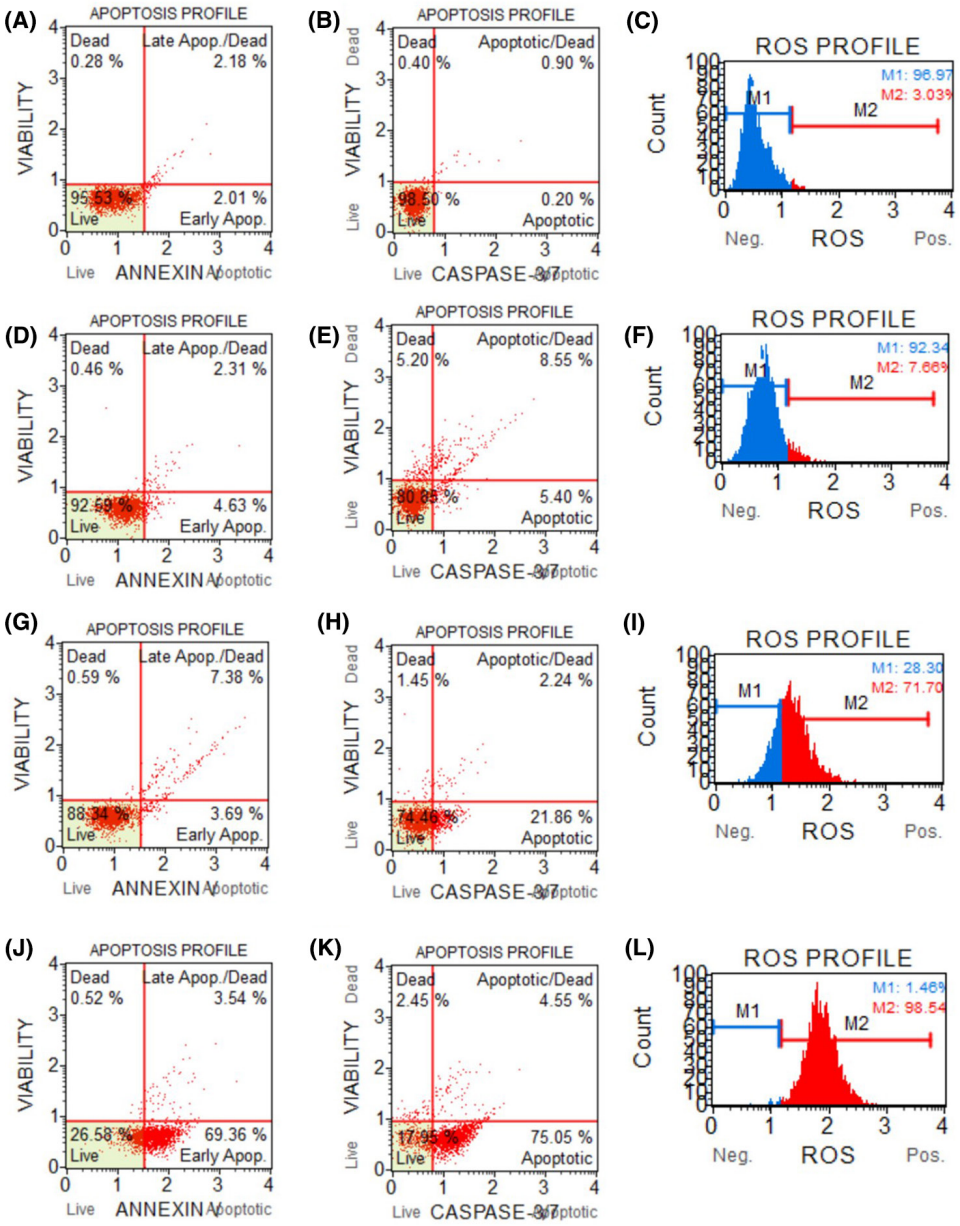
Annexin V assay (A, D, G, J), caspase 3 and 7 assay (B, E, H, K) and oxidative stress (C, F, I, L) results of radiated riboflavin‐applied C6 cell line. (A), (B) and (C) are control; (D), (E) and (F) are 31.25 μM of radiated riboflavin treated; (G), (H) and (I) are 62.5 μM of radiated riboflavin‐treated and (J), (K) and (L) are 93.75 μM of radiated riboflavin‐treated cells. In (F), (I) and (L), M1 (blue) and M2 (red) shows ROS negative and ROS positive cell populations, respectively.

Regarding the apoptotic test, as depicted in Figure 2A,D,G,J, in control cells, the overall viability was 95.53%. The overall ratio of cell apoptosis was found to be 4.19%, consisting of 2.01% early apoptotic cells and 2.18% late apoptotic cells. In radiated riboflavin‐administered cells, at the minimum administered dosage (31.25 μM), the cellular viability decreased to 92.59%, overall apoptotic cells were recorded as 6.94% with an early‐stage apoptotic cells of 4.63% and late‐stage apoptotic cells of 2.31%. After the intermediate concentration treatment (62.5 μM), the cellular viability diminished to 88.34%. The overall apoptotic cells was recorded as 11.07% with an early apoptosis of 3.69% and late apoptosis of 7.38. Finally, at the maximum concentration (93.75 μM), the cellular viability decreased to 26.58%. In addition, total apoptosis was recorded as 72.9% with an early apoptosis of 69.36% and late apoptosis of 3.54.

### 3.3 Caspase 3/7 test findings

Regarding the caspase 3/7 results, as depicted in Figure 2B,E,H,K, in control cells, the overall viability was 98.50%. The overall ratio of cell apoptosis was found as 1.1%, consisting of 0.2% early apoptotic cells and 0.9% late apoptotic cells. In radiated riboflavin‐administered cells, at the minimum administered dosage (31.25 μM), the viability of the cells decreased to 80.88%, overall apoptotic cells were recorded as 13.95 with an early apoptosis of 5.40% and late apoptosis of 8.55%. At the intermediate concentration (62.5 μM), the viability decreased to 74.46%. The overall ratio of cell apoptosis was recorded as 24.1% with an early apoptosis of 21.86% and late apoptosis of 2.24. Eventually, at the maximum applied concentration (93.75 μM), the viability decreased to 17.95%. In addition, total apoptosis was recorded as 79.6% with an early apoptosis of 75.05% and late apoptosis of 4.55.

### 3.4 Findings on oxidative stress

Regarding the oxidative stress findings, as depicted in Figure 2C,F,I,L, only 3.03% of the control cells are ROS positive. 31.25 μM of radiated riboflavin increased ROS positive cells to 7.66%. However, 62.5 μM of radiated riboflavin induced ROS in more than 70% of the cells. What's more, after 93.75 μM of radiated riboflavin was administered, almost all cells showed ROS positivity.

### 3.5 The results of inflammation‐associated cytokines

**FIGURE 3:**
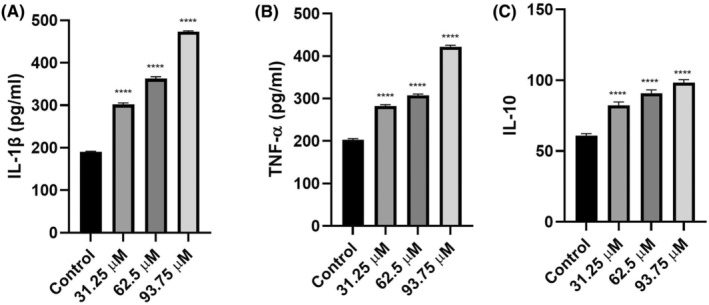
IL‐1β (A), TNF‐α (B) and IL‐10 (C) levels of radiated riboflavin‐applied C6 cell line. ^****^ *p* < 0.0001 in comparison to the control amounts. The outcomes are deemed as the average ± SD.

IL‐1β levels augmented with ascending doses of radiated riboflavin. With increased doses of radiated riboflavin, IL‐1β levels increased 1.5, 1.9 and 2.5 times the control levels. All radiated riboflavin‐applied cells were significantly different than untreated cells (*p* < 0.0001, Figure 3A). The minimum average amount of IL‐1β was 192.2 pg/mL in the untreated cells, and the maximum average amount was 473.1 pg/mL in the 93.75 μM‐radiated riboflavin treated cells.

TNF‐α levels augmented with ascending doses of radiated riboflavin. With increased doses of radiated riboflavin, TNF‐α levels increased 1.4, 1.5 and 2.1 times the control levels. All radiated riboflavin‐applied cells were significantly different than untreated cells (*p* < 0.0001, Figure 3B). The minimum average amount of TNF‐α was 203.3 pg/mL in the untreated cells, and the maximum average amount was 421.8 pg/mL in the 93.75 μM‐radiated riboflavin treated cells.

IL‐10 levels increased with ascending doses of radiated riboflavin. With increased doses of radiated riboflavin, IL‐10 levels increased 1.35, 1.5 and 1.62 times the control levels. All radiated riboflavin‐applied cells were significantly different than untreated cells (*p* < 0.0001, Figure 3C). The minimum average amount of IL‐10 was 60.8 pg/mL in the untreated cells, and the maximum average amount was 98.3 pg/mL in the 93.75 μM‐radiated riboflavin treated cells.

### 3.6 Caspase 3 and caspase 9 findings

**FIGURE 4:**
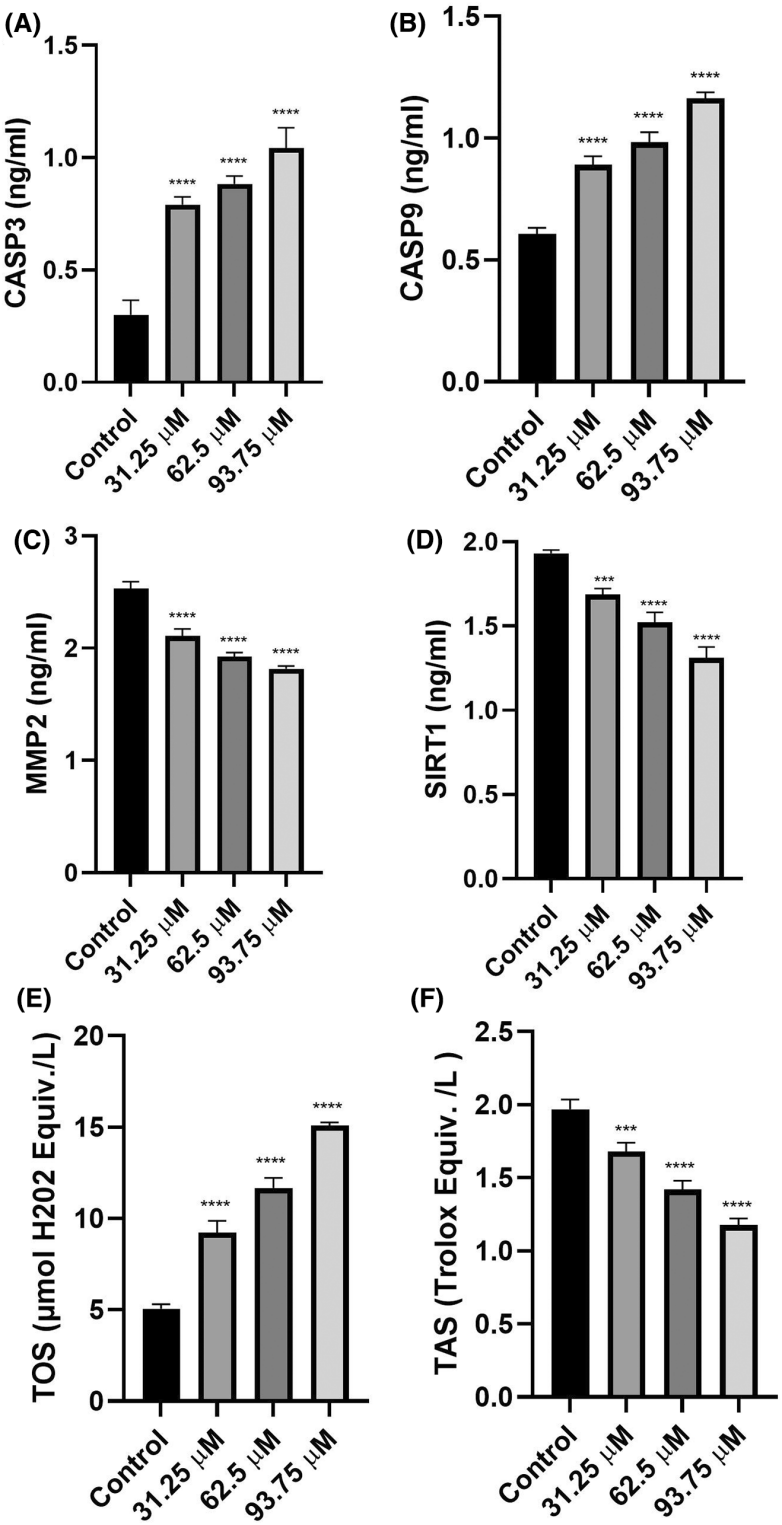
CASP3 (A), CASP9 (B), MMP2 (C), SIRT1 (D), TOS (E) and TAS (F) levels of radiated riboflavin‐administered C6 cell line. ***p < 0.001 and ****p < 0.0001 relative to the untreated cells. The findings are deemed as the average ± SD.

The CASP3 levels climbed up with ascending doses of radiated riboflavin. In comparison to the control amounts, caspase 3 amounts rose up to 2.5, 3 and 3.5 times with increased doses of radiated riboflavin. All radiated riboflavin‐applied cells were significantly different than untreated cells (*p* < 0.0001, Figure 4A). The minimum average amount of CASP3 was 0.30 ng/mL in the nontreated cells, and the maximum average amount was 1.04 ng/mL in the 93.75 μM‐radiated riboflavin treated cells.

The CASP9 levels climbed up with the ascending doses of radiated riboflavin. In comparison to the control amounts, CASP3 amounts rose up to 1.46, 1.6 and 1.9 times with increased doses of radiated riboflavin. All radiated riboflavin‐applied cells were significantly different than untreated cells (*p* < 0.0001, Figure 4B). The minimum average amount of CASP3 was 0.61 ng/mL in the nontreated cells, and the maximum average amount was 1.16 ng/mL in the 93.75 μM‐radiated riboflavin treated cells.

### 3.7 MMP2 results

The MMP2 levels decreased with the ascending doses of radiated riboflavin. In comparison to the control levels, MMP2 levels decreased by almost 28% at the highest radiated riboflavin dose. All radiated riboflavin‐applied cells were significantly different than untreated cells (*p* < 0.0001, Figure 4C). The minimum average amount of MMP2 was 1.82 ng/mL in the 93.75 μM‐radiated riboflavin treated cells, and the maximum average amount was 2.53 ng/mL in the untreated cells.

### 3.8 SIRT1 findings

The SIRT1 amounts decreased with the ascending doses of radiated riboflavin. In comparison to the control amounts, SIRT1 amounts decreased by almost 33% at the highest radiated riboflavin dose. All radiated riboflavin‐applied cells were significantly different than untreated cells (*p* < 0.0001, Figure 4D). The minimum average amount of SIRT1 was 1.31 ng/mL in the 93.75 μM‐radiated riboflavin treated cells, and the maximum average amount was 1.93 ng/mL in the not‐treated cells.

### 3.9 TOS and TAS results

TOS levels rose with ascending doses of radiated riboflavin. With increased doses of radiated riboflavin, TOS levels increased 1.8, 2.3 and 3.0 times the control levels. All radiated riboflavin‐applied cells were significantly different than untreated cells (*p* < 0.0001, Figure 4E). The minimum average amount of TOS was 5.06 μmol H_2_0_2_ Equiv./L in the untreated cells, and the maximum average amount was 15.1 μmol H_2_0_2_ Equiv./L in the 93.75 μM‐radiated riboflavin treated cells.

TAS levels decreased with the ascending doses of radiated riboflavin. In comparison to the control levels, TAS levels decreased by almost 40.3% at the highest radiated riboflavin dose. All radiated riboflavin‐applied cells were significantly different than untreated cells (*p* < 0.0001, Figure 4F). The minimum average amount of TAS was 1.18 Trolox equiv./L in the 93.75 μM‐radiated riboflavin treated cells, and the maximum average amount was 1.97 Trolox equiv./L in the control cells.

## 4 DISCUSSION

In the present study, we sought to ascertain the impact of varying radiated and nonradiated riboflavin concentrations on C6 cell line. We conducted MTT cytotoxicity assays for 24, 48 and 72 h. Since we detected a more significant antiproliferative effect of radiated riboflavin than nonradiated one, we move forward our subsequent experiments with radiated one. We carried out biochemical, fluorometric and apoptotic assays.

Based on the MTT cytotoxicity assay IC50 of radiated riboflavin in C6 cells were revealed as 88.01 μM, 76.85 μM and 57.60 μM for 24, 48 and 72 h of treatments, respectively. We could not properly detect the IC50 concentrations of nonradiated riboflavin since the alleged proliferative effects of the substance, which gives an increasing time‐course IC50 concentrations. Although the first 24‐h experiment, there was a consistent, dose‐dependent proliferative effect of nonradiated riboflavin, this converted to proliferative effects on the longer time treatments. In addition, we could not determine the all‐cells‐killing dose of nonradiated riboflavin at our experimental conditions. In even the highest doses of nonradiated riboflavin, some viability was detected by MTT assay. Many plausible interpretations can be deduced from these findings. It can be directly interpreted as the proliferative effects of nonradiated riboflavin. Proliferative effects of riboflavin (nonradiated) are documented in the study of Yang et al. (2013) in three lung cancer cells, especially higher than 200 μM, and which was more apparent after 72 treatments, with control cells having no proliferation.^14^ A previous investigation revealed the sensitivity of B16F10 melanoma cells to 50 μM irradiated riboflavin, as indicated by MTT analysis results.^15^ Furthermore, the researchers identified IC50 values of 80 μM and 35 μM following the treatment of melanoma cells with irradiated riboflavin for 12 and 48 h, respectively. In addition, this study demonstrated that irradiated riboflavin leads to a 50% reduction in BrdU incorporation in melanoma cells. This effect correlates with a diminished proliferation rate and the induction of cell cycle arrest or cell death, consequently leading to a reduction in the number of colonies. Consistently, we also detected a proliferative effect of nonradiated riboflavin especially at the doses of 62.5 and 125 μM for 72 h treatment. Alternatively, although less probable, it is conceivable that this proliferation can be because either the MTT dye could interfere with riboflavin, or an overwhelming cell population might influence the outcomes. Nevertheless, it remains indisputable that radiated riboflavin significantly and more effectively reduces C6 cell viability compared to its nonradiated counterpart.

Riboflavin was found to have tumour‐inhibiting effects on pro‐monocytic lymphoma cells.^3^ In the same article, apoptosis or cell death by necrosis were not observed after riboflavin treatment (250 μg/mL ~ 665 μM); the distribution of live and dead cells was comparable to that of untreated cells. And migration of the cells is reported to be decreased after riboflavin treatment. And IL‐10 levels increased as in our experiment (0.125 g/mL). The doses used in this study were very low range. And the IC50 dose is not clearly observed in the graph, all doses used seem to either increase or maintain proliferation stable.^3^

Cumulative evidence suggests that a strong linkage exists in the triangle of oxidative stress, chronic inflammation and cancer.^16^ Indeed, oxidative stress is a two‐edged sword regarding cancer. It exerts both pro‐ and anti‐tumourigenic actions. On one hand, the onset and progression of cancer have been associated with oxidative stress‐induced DNA damage, genomic instability and cell proliferation. On the other hand, it leads to senescence and apoptosis, counteracting tumour growth.^17^ In our experimental observations, both biochemical and flow cytometric measurements revealed an elevation in total oxidative status. Additionally, a concomitant decrease in total antioxidants was noted.

Due to their crucial roles in several processes involved in tumour metastasis, such as tumour‐induced angiogenesis, tumour invasion and the development of metastatic foci at secondary locations, MMPs have garnered a lot of attention. The abundance of evidence that has been gathered to support the importance of MMPs in these processes highlights their potential as therapeutic targets.^18^ In one study, the impacts of irradiated riboflavin on androgen‐independent human prostate cancer cells were explored.^11^ Riboflavin photoproducts were suggested to be cytotoxic to the cells through FasL–Fas signalling. More importantly, they pointed out that irradiated riboflavin suppressed matrix degrading proteases, leading to expression of VEGF and inhibition of TIMP1, indicating an antimetastatic activity. Consistently, also in our study, we noted a dose‐dependent decrease (28% decrease at highest dose) in MMP2 activities (one of the metalloproteinases). It is conceivable to say that radiated riboflavin not only boost cell death, particularly apoptosis but also demonstrated inhibitory effects on metalloproteinases and ruling out the putative proliferative effects of nonradiated riboflavin. Conversely, nonradiated riboflavin was reported to boost MMP levels.^14^ In addition, in the same study, they claim that irradiated riboflavin exerts a cell specific toxicity. In their MTT reduction graph, it is apparent that while radiated riboflavin up to 50 μM posed a substantial antiproliferative effects on PC3 cells but not human hepatocytes and rat prostate smooth cells. And approximate IC50 for 24 h exposure is 35 μM < IC50 < 40 μM according to our interference from graphical representation. This effective dose for 24 h treatment is higher than our 24 h‐IC50 dose, which is 88 μM; however, they are close to each other. This disparity can stem from the resistance of C6 cells to radiated riboflavin more than PC3 cells if we rule out the possible disparities or variation between experimental conditions and other factors. If radiated riboflavin shows a cell specific effects, as claimed in the above article, it makes sense to check on the effect of this substance on normal glia cells and compare it with malignant glioblastoma cells in future experiments.

The Sirtuin family constitutes a group of nuclear‐encoded mitochondrial proteins.^19^ Specifically, SIRT1, an NAD‐dependent protein deacetylase, plays a pivotal role in various essential biological processes, encompassing energy metabolism, DNA repair and mitochondrial homeostasis.^20^ These processes are integral for cell growth, differentiation, migration and survival, rendering SIRT1 a critical regulator implicated in numerous diseases.^21^ SIRT1's involvement in cancer has been defined by dichotomous roles, demonstrating dual effects as both a tumour suppressor and an oncogene depending on cancer type and stage.^22^ In a prior study, tissue analysis revealed the overexpression of SIRT1 in glioma tissues in comparison to nontumour tissues.^23^ Similarly, in the study of Li et al (2019), it is indicated that glioma tissues had higher levels of SIRT1 expression relative to nearby brain tissues. SIRT1 was proposed to be affirmatively linked with cell viability, proliferative ability and growth of U87 glioblastoma cells.^24^ Furthermore, it has been reported that the tumour suppressor p53 exerts a downregulating effect on SIRT1 expression in glioma tumour cells.^25^ Additionally, SIRT1 has been shown to induce inactivation of p53 and inhibit p53‐dependent apoptosis.^26^ Moreover, SIRT1 has been implicated in promoting the viability of glioma tumour cell lines while concurrently inhibiting apoptosis.^27^ In this study, we identified a dose‐dependent diminish in SIRT1 levels, exhibiting the ability of irradiated riboflavin suppressing C6 glioblastoma cells proliferation through SIRT1 signalling. Additionally, the assessment of apoptotic status employed two distinct methodological approaches in our study: biochemical and flow cytometric assays. In the biochemical measurements, we identified a dose‐dependent escalation in caspase 3 and 9 activities. Furthermore, a dose‐dependent increase was observed in both Annexin V and caspase 3 and 7 assays.

In the present study, radiated riboflavin increased both pro‐inflammatory cytokines IL1β and TNF‐α and anti‐inflammatory cytokine IL‐10. The dramatic early increase in IL‐10, canonically classified as an anti‐inflammatory cytokine, appears to be a hallmark of hyperinflammatory signalling responses of the cells to compensate this sudden hyperinflammation. Also, this contradictory increase of both pro‐ and anti‐inflammatory cytokines can be also explained by mix activation of M1 proinflammatory and M2 anti‐inflammatory microglia, each of which releases functionally opposite cytokines^18^ because C6 glioblastoma cell lines can have mixture of those glial cells.

To sum up, we have demonstrated that irradiated riboflavin has superior effects on C6 glioblastoma cells compared to nonradiated riboflavin in terms of apoptosis, antiproliferation, oxidative stress and the activation of certain signalling pathways. Nonradiated riboflavin shows the potential to enhance the growth of C6 glioblastoma cells, and this effect may extend to other types of cancer cells as well. However, further studies are warranted to shed light on these pathways and ascertain the differences between irradiated and nonradiated riboflavin.

## DISCLAIMER

These results do not show a clinical relevance, just show an idea that radiated riboflavin has better effects over C6 cells than nonradiated one and riboflavin might show proliferative effect, if the results do not stem from any confounding factors like assay interference or any other. These doses can only give an idea for researchers to do further research, and used as an advice or recommendation for clinical research.

## AUTHOR CONTRIBUTIONS

**Sedat Kacar:** Conceptualization (equal); data curation (equal); formal analysis (equal); funding acquisition (equal); investigation (equal); methodology (equal); project administration (equal); resources (equal); software (equal); supervision (equal); validation (equal); visualization (equal); writing – original draft (lead); writing – review and editing (equal). **Ceyhan Hacioglu:** Conceptualization (equal); data curation (equal); formal analysis (equal); funding acquisition (equal); investigation (equal); methodology (equal); project administration (equal); resources (equal); software (equal); supervision (equal); validation (equal); visualization (equal); writing – original draft (equal); writing – review and editing (equal). **Fatih Kar:** Formal analysis (equal); methodology (equal).

## FUNDING INFORMATION

The authors declare that no funds were received during the preparation of this manuscript.

## CONFLICT OF INTEREST STATEMENT

All authors declare that there are no conflicts of interest.

## Data Availability

The datasets generated during and/or analysed during the current study are available from the corresponding author on reasonable request.
